# Fasting as Medicine: Mitochondrial and Endothelial Rejuvenation in Vascular Aging

**DOI:** 10.1111/acel.70372

**Published:** 2026-01-11

**Authors:** Madison Milan, Eva Troyano‐Rodriguez, Jennifer Ihuoma, Sharon Negri, Rakesh Rudraboina, Aleksandra Kosmider, Shantipriya Awasthi, Priya Balasubramanian, Shannon Conley, Andriy Yabluchanskiy, Anna Csiszar, Zoltan Ungvari, Rafael de Cabo, Stefano Tarantini

**Affiliations:** ^1^ Vascular Cognitive Impairment and Neurodegeneration Program, Reynolds Oklahoma Center on Aging/Center for Geroscience and Healthy Brain Aging, Department of Neurosurgery University of Oklahoma Health Sciences Center Oklahoma City Oklahoma USA; ^2^ Stephenson Cancer Center University of Oklahoma Health Sciences Center Oklahoma City Oklahoma USA; ^3^ Oklahoma School of Science and Math Oklahoma City Oklahoma USA; ^4^ Department of Cell Biology University of Oklahoma Health Sciences Center Oklahoma City Oklahoma USA; ^5^ International Training Program in Geroscience, Doctoral School of Basic and Translational Medicine/Department of Public Health Semmelweis University Budapest Hungary; ^6^ Department of Health Promotion Sciences, College of Public Health University of Oklahoma Health Sciences Center Oklahoma City Oklahoma USA; ^7^ Translational Gerontology Branch, Intramural Research Program, National Institute on Aging, National Institutes of Health Baltimore Maryland USA

**Keywords:** aging, neuroinflammation, neuroprotection, nutritional interventions, oxidative stress

## Abstract

Aging drives a progressive decline in vascular health, undermining endothelial function, neurovascular coupling (NVC), and blood–brain barrier (BBB) integrity, three processes essential for maintaining cerebral perfusion and cognitive resilience. Central to these age‐related deficits is mitochondrial dysfunction, which disrupts redox balance, bioenergetics, and nutrient‐sensing pathways within vascular cells, thereby promoting oxidative stress, impaired mitophagy, mitochondrial fragmentation, and endothelial senescence. These molecular derangements are especially consequential in the brain's microvasculature, where the exquisite metabolic demands of neural tissue depend on intact endothelial signaling. As a result, cerebrovascular aging becomes a major driver of cognitive decline and vascular contributions to dementia. This review synthesizes current mechanistic insights into mitochondrial and endothelial pathways that shape vascular aging, with particular focus on the neurovascular unit. We further highlight emerging evidence that time‐restricted feeding/eating (TRF/TRE), a circadian‐aligned dietary intervention that limits food intake to a daily feeding window without reducing calories, can restore mitochondrial function, activate adaptive nutrient‐sensing networks including AMPK and SIRT1, suppress mTOR signaling, and promote metabolic switching toward ketone synthesis and utilization. Through these mechanisms, TRF enhances endothelial resilience, preserves NVC and BBB integrity, and may counteract the cerebrovascular processes that accelerate cognitive aging. Understanding how TRF/TRE re‐engages mitochondrial and vascular repair programs offers a translational framework for developing accessible, non‐pharmacological strategies to extend healthspan and mitigate age‐related cognitive impairment.

## Graphical abstract

Aging impairs cerebrovascular health by driving mitochondrial dysfunction, oxidative stress, endothelial failure, and neurovascular uncoupling, leading to BBB breakdown and cognitive decline. In contrast, time‐restricted feeding/eating counteracts these mechanisms by restoring mitochondrial function, activating adaptive nutrient‐sensing pathways, preserving neurovascular coupling, maintaining BBB integrity, and promoting cerebrovascular rejuvenation and cognitive resilience.

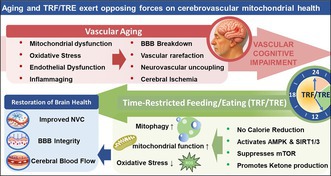

## 1 Introduction

As global lifespan continues to rise, with individuals over 60 projected to reach 1.4 billion by 2030 and life expectancy in the United States now exceeding 77 years, aging is the strongest risk factor for cardiovascular disease, cerebrovascular dysfunction, and dementia (Azam et al. 2021; Xia et al. 2018). This reflects a progressive erosion of vascular resilience across multiple organ systems (Ageing and health 2021) (Kochanek et al. 2022). Nowhere is this decline more consequential than in the brain, where the integrity of the microvasculature governs oxygen delivery, metabolic support, neurovascular coupling (NVC), and the maintenance of the blood–brain barrier (BBB) (Ihuoma et al. 2025; Patai, Csik, et al. 2025; van Dinther et al. 2024). Even subtle impairments in endothelial cell (EC) function can disrupt cerebral perfusion and accelerate cognitive decline (Gulej et al. 2024; Nyul‐Toth, Patai, et al. 2024; Waigi et al. 2024). Thus, understanding why the vasculature fails with age, and how to intervene upstream—remains central to efforts to extend healthspan and preserve cognitive function (Garmany et al. 2021). A growing body of work identifies mitochondrial dysfunction as a unifying driver of vascular aging. Across arteries, arterioles, and capillaries, aging shifts mitochondrial biology toward reduced oxidative phosphorylation, increased reactive oxygen species (ROS) production, impaired mitophagy, and dysregulated nutrient sensing. These changes amplify endothelial senescence, diminish nitric oxide (NO)‐mediated vasodilation, and promote neuroinflammation and BBB breakdown. In the brain, where metabolic demands are exceptionally high, mitochondrial decline leads to neurovascular uncoupling, reduced cerebral blood flow (CBF), and a heightened susceptibility to both Alzheimer's disease (AD) and vascular dementia (VaD) (Nyul‐Toth, Patai, et al. 2024; Waigi et al. 2024). Mitochondrial deterioration within ECs therefore represents a critical point of failure linking systemic aging to neurodegeneration. Despite this vulnerability, the aging vasculature retains a remarkable degree of plasticity. Interventions that enhance mitochondrial quality control, restore redox balance, or recalibrate nutrient‐sensing pathways, such as SIRT1, AMPK, and mTOR, have shown robust benefits in preclinical models. Among these, time‐restricted feeding (TRF) in rodents and time‐restricted eating (TRE) in humans, which will together be referred to as “TRF/TRE” from here on, has emerged as a particularly promising and feasible lifestyle intervention. Unlike chronic caloric restriction, TRF/TRE restricts food intake to a consistent daily window without reducing total caloric consumption. Yet TRF/TRE recapitulates many of the metabolic, vascular, and cognitive benefits of caloric restriction, including improved endothelial function, enhanced mitochondrial respiration, increased mitophagy, reduced oxidative stress, and improved NVC. Crucially, TRF/TRE induces a metabolic switch from glucose dependence to increased fatty acid oxidation and ketone utilization, a state that favors mitochondrial efficiency, enhances antioxidant defenses, and reduces systemic inflammation. Ketone bodies such as β‐hydroxybutyrate also function as signaling molecules that modulate histone deacetylation, promote angiogenesis, and suppress endothelial senescence. These metabolic changes converge on the same pathways dysregulated by aging, offering a mechanistic rationale for TRF/TRE as a strategy to restore vascular homeostasis. In this review, we synthesize emerging evidence linking mitochondrial dysfunction to vascular aging and describe how TRF/TRE targets these processes at multiple levels of the neurovascular unit. We highlight the mitochondria–endothelial axis as a central determinant of cerebrovascular resilience and evaluate the potential of TRF/TRE to rejuvenate vascular function, preserve BBB integrity, and protect cognitive health. By integrating mechanistic, preclinical, and translational insights, we aim to provide a unified framework for understanding how fasting‐based interventions may counteract vascular aging and extend healthspan. Prior reviews have emphasized the systemic metabolic and endocrine benefits of intermittent fasting and TRF/TRE. In contrast, this review specifically integrates endothelial mitochondrial biology, neurovascular unit (NVU) function, and TRF/TRE, highlighting vascular mechanisms as central drivers of cognitive aging. By positioning the mitochondria–endothelium axis at the heart of both vascular decline and fasting‐mediated rejuvenation, we aim to provide a unified mechanistic framework for understanding how TRF/TRE may preserve cerebrovascular integrity, maintain cognitive resilience, and extend healthspan.

## 2 Main Text

In the following sections, we systematically examine the pathophysiology of vascular aging, dissect the central role of mitochondrial dysfunction in endothelial cells, and evaluate how TRF/TRE offers a promising strategy to restore cerebrovascular health and preserve cognitive resilience.

## 3 The Vascular Aging Phenotype: A Mitochondrial–Endothelial Framework

**FIGURE 1:**
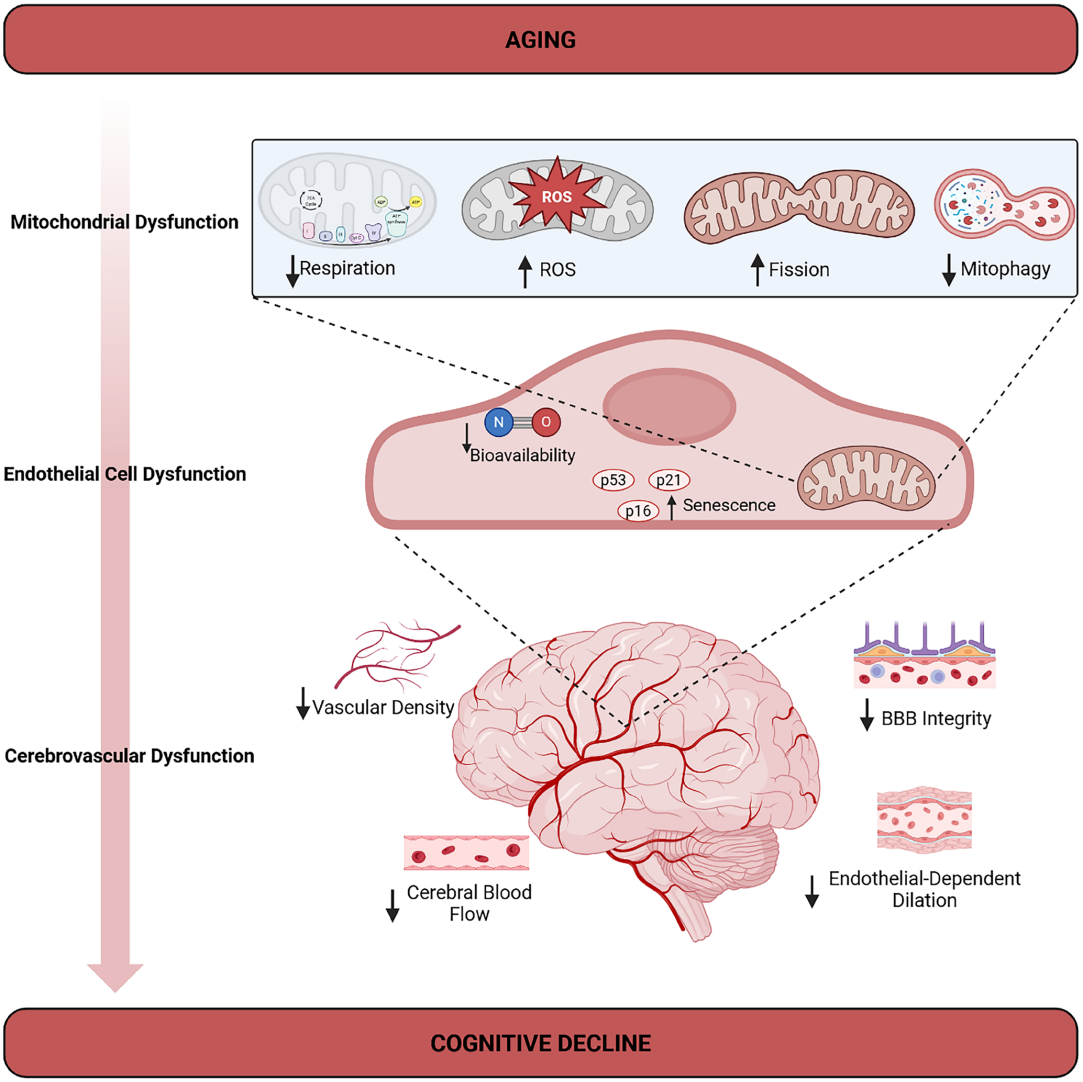
Mitochondrial dysfunction is a driver of cerebrovascular aging. Mitochondrial dysfunction is an established hallmark of aging. This can include decreased mitochondrial respiration, increased ROS production, increases in fission and mitochondrial fragmentation, and decreased mitophagy. These occurrences within the mitochondria of ECs can impact the overall cellular health, including decreases in tight junction integrity and NO availability and increases in endothelial senescence. Ultimately, these changes can lead to cerebrovascular physiological changes during aging which are characterized by loss of BBB integrity and decreased EDD, CBF, and vascular density. These alterations can result in cognitive decline.

The global expansion of older populations is accompanied by a disproportionate surge in disorders driven by vascular failure, such as vascular cognitive impairment (VCI) (Jost and Kujach 2025). Cardiovascular disease remains the leading global cause of death, and ischemic heart disease has ranked as the top cause of years of life lost worldwide for the last decade (Toth et al. 2017). In parallel, dementia affects ~55 million people today and is projected to reach ~139 million by 2050, with VaD, VCI, and mixed dementias accounting for a substantial fraction of cases (Csipo et al. 2025; Shin 2022; Ungvari et al. 2024). Within the “hallmarks of aging” framework, vascular aging sits at the intersection of multiple hallmarks (Lopez‐Otin et al. 2013), including mitochondrial dysfunction (Qu et al. 2022; Quintero et al. 2006), impaired intercellular communication (Sena et al. 2018), and cellular senescence (Han and Kim 2023; Li, Yang, et al. 2024) and acts as a systems‐level driver of organ failure, particularly in the brain (Ungvari, Tarantini, Donato, et al. 2018; Lopez‐Otin et al. 2023). With age, mitochondrial performance in vascular cells declines: respiratory chain efficiency decreases, ROS production increases, mitochondrial dynamics are disrupted, mitophagy falters, and mtDNA damage accumulates (Bondy 2024; Jedlicka et al. 2022; Kim et al. 2018; Tyrrell et al. 2020; Zhu et al. 2023). These changes establish a cascade of dysfunction that spans vascular beds, including cerebral microvasculature, and promote endothelial senescence, impaired vasodilatory capacity, NVC, and BBB breakdown (Figure 1). Here, we frame the vascular aging phenotype as the emergent consequence of a dysfunctional mitochondrial–endothelial axis. We focus on (i) systemic endothelial dysfunction and arterial remodeling, (ii) cerebrovascular rarefaction and neurovascular uncoupling, and (iii) BBB and NVU breakdown, and show how mitochondrial defects in endothelial cells (ECs) integrate these processes into a self‐reinforcing network.

### 3.1 Systemic Endothelial Dysfunction and Arterial Remodeling

Across the arterial tree, aging is characterized by a stereotyped shift from a highly adaptive, nitric oxide (NO) dominated vasculature to one marked by oxidative stress, inflammation, and stiffness. ECs in young vessels maintain vascular tone, hemostasis, and immune quiescence by producing NO via endothelial nitric oxide synthase (eNOS), integrating signals from shear stress, calcium–calmodulin, and cofactors such as tetrahydrobiopterin (BH_4_). With advancing age, endothelial dysfunction can be driven by several factors, with oxidative stress and inflammation being among the most prominent. Several studies reported an overall decrease in mitochondrial respiration during aging (Jedlicka et al. 2022; Rosa et al. 2023), including arterial and brain vessels; complex I activity is particularly vulnerable. Defects in electron‐transport kinetics lead to increased electron leakage and superoxide (O_2_•^−^) generation. Increased levels of hydrogen peroxide (H_2_O_2_) and O_2_•^−^ in the vasculature exacerbate decline in NO bioavailability (Ungvari, Tarantini, Donato, et al. 2018). A key mechanistic pivot in aging is the mitochondrial generation of reactive oxygen species (mtROS), which react rapidly with NO to form peroxynitrite (ONOO^−^). This both scavenges NO and oxidizes BH_4_, driving “eNOS uncoupling” such that eNOS itself becomes a source of superoxide rather than NO (Dichgans and Leys 2017). This rise in mtROS not only damages mitochondrial lipids, proteins, and mtDNA but also shifts the endothelial redox state toward oxidative stress and promotes endothelial dysfunction (Kirkman et al. 2021). For example, studies of aging vessels show augmented 4‐hydroxynonenal and malondialdehyde levels alongside lower superoxide dismutase (SOD) and catalase activity, indicating impaired antioxidant defenses (Gioscia‐Ryan et al. 2014). This can lead to the low chronic levels of inflammation that have been observed in the aging vasculature (Rodriguez‐Manas et al. 2009), alongside increases in vasoconstrictors such as endothelin‐1 (ET‐1), thromboxane A_2_, and angiotensin II (Dichgans and Leys 2017). Together, these changes shift the vascular redox state toward oxidative stress and promote endothelial dysfunction, even in the absence of overt atherosclerotic plaque. Concurrently, structural remodeling of large arteries, elastin fragmentation, collagen deposition, and calcification leads to arterial stiffening and widened pulse pressure (Akhiyat et al. 2025). Loss of Windkessel function transmits excessive pulsatile energy into the microcirculation, especially in high‐flow, low‐resistance beds such as the brain and kidney (Armstrong et al. 2024; Chirinos et al. 2019; Lin et al. 2025). This hemodynamic insult exacerbates microvascular rarefaction, damages the endothelial glycocalyx, and further promotes NO/ET‐1 imbalance. From a systems perspective, mitochondrial dysfunction in ECs is not an isolated lesion but a nodal alteration that couples redox stress, mechanical load, and inflammatory signaling to produce the classic macro‐ and microvascular features of vascular aging (Tang et al. 2014).

### 3.2 Cerebral Microvascular Rarefaction and Neurovascular Uncoupling

The brain is uniquely vulnerable to vascular aging. It accounts for ~2% of body weight but ~20% of resting oxygen consumption and depends on tight spatiotemporal matching between neuronal activity and blood flow, termed NVC (Iadecola 2017). During aging, the brain experiences a decline in cortical and hippocampal microvascular density, shortened vessel length (Bennett et al. 2024; Nyul‐Toth et al. 2021), reduced branching of cerebral blood vessels (Bennett et al. 2024; Chandragiri et al. 2025; Negri et al. 2025; Nyul‐Toth, Negri, et al. 2024; Pinckard et al. 2025), and increased arterial stiffness in both rodent models and humans (Herzog et al. 2025). Together, these vascular changes contribute to decreased CBF and reduced microvascular density (Negri et al. 2025; Nyul‐Toth, Negri, et al. 2024; Walker et al. 2023). Increased stiffness of arteries is associated with cognitive impairment (Tsao et al. 2013), and more specifically, age‐related aortic stiffness and consequent elevation in pulse pressure may lead to microvascular rarefaction (Tsao et al. 2013). At the cellular level, ECs in cerebral microvessels exhibit many of the same mitochondrial alterations described systemically (Milan et al. 2024), decreased respiratory chain capacity, increased mtROS, and impaired mitophagy (Grossini et al. 2025). Healthy mitochondrial networks constantly undergo fission and fusion, enabling exchange of contents, removal of damaged segments, and maintenance of mitochondrial integrity. In aging ECs, mitochondrial dynamics shift toward excessive fission, driven by increased expression of dynamin‐related protein 1 (Drp1) and mitochondrial fission 1 protein (Fis1), and away from fusion, as reflected by reduced levels of mitofusin 2 (Mfn2) and optic atrophy protein 1 (Opa1), leading to fragmented mitochondria that are less capable of ATP generation and more prone to ROS emission (Chen et al. 2023; Yapa et al. 2021; Zhu et al. 2023). In parallel, mitophagy (the selective autophagic elimination of mitochondria) becomes less efficient with age, enabling accumulation of malfunctioning mitochondria and further redox stress (Fivenson et al. 2017). In parallel, Han et al. (2024) demonstrated that impaired mitophagy in aged vascular endothelial cells results in accumulation of damaged mitochondria, heightened mtROS production, and exacerbated vascular inflammation. Mitochondrial biogenesis also declines with age, in large part because peroxisome proliferator‐activated receptor gamma coactivator‐1 alpha (PGC‐1α) and sirtuin‐1 (SIRT1) become downregulated, limiting the capacity of endothelial cells to renew their mitochondrial pool.

Consistent with this evidence, experimental manipulation of mitochondrial fission–fusion balance, mitophagy, or the PGC‐1α–SIRT1 biogenesis axis in endothelial and vascular cells causally alters endothelial senescence, NO bioavailability, and vasodilator function. In this context, small changes in NO signaling or energy supply translate rapidly into NVC failure. In the healthy NVU, glutamatergic synaptic activity elevates intracellular Ca^2+^ in neurons and astrocytes, triggering production of NO, prostaglandins (PGE_2_), and epoxyeicosatrienoic acids (EETs) that relax vascular smooth muscle cells (VSMCs) and pericytes to increase local CBF (Gemsenjager 1990). Aging blunts these responses: human studies using functional near‐infrared spectroscopy (fNIRS), transcranial Doppler, or retinal dynamic vessel analysis show reduced stimulus‐evoked vasodilation with advancing age and in individuals with MCI and early dementia (Beishon et al. 2021; Peterfi et al. 2024). Preclinical work links these NVC deficits directly to endothelial mitochondrial redox status. In vivo 2‐photon imaging demonstrated that age‐related mitochondrial oxidative stress in cerebral endothelial cells diminishes NO–dependent NVC responses and contributes to cognitive deficits (Csik et al. 2025; Tarantini et al. 2015). In aged mice, genetic or pharmacological reduction of mtROS (e.g., mitochondrial catalase or mitochondrial‐targeted antioxidants such as MitoQ or SS‐31) restores NO‐mediated vasodilation and rescues NVC, with parallel improvements in spatial learning and working memory (Dhanekula et al. 2025; Dichgans and Leys 2017; Patai, Patel, et al. 2025; Tarantini et al. 2018). Conversely, experimental elevation of ROS in young animals via redox‐cycling agents is sufficient to impair NVC and cognitive performance, phenocopying aspects of cerebrovascular aging (Xu et al. 2017). These studies support a causal model in which endothelial mitochondrial dysfunction acts upstream of NVC impairment and cognitive decline, positioning the endothelium as both a sensor and effector of brain aging.

### 3.3 Blood–Brain Barrier and Neurovascular Unit Breakdown

The BBB is not simply a physical barrier, but a dynamic vascular interface maintained by mitochondrial‐competent ECs, pericytes, and astrocyte end feet (Kaya and Ahishali 2021). In aging, mitochondrial dysfunction in ECs contributes to BBB breakdown: oxidative damage to tight‐junction proteins (claudin‐5, ZO‐1), increased paracellular permeability, and altered transporter activity (GLUT1, MCT1) are observed (Wang, Chen, et al. 2025; Zhang et al. 2025). This results in impaired nutrient delivery and toxin clearance (Knox et al. 2022; Nyul‐Toth et al. 2021), alongside the leakage of plasma proteins, immune mediators, and metabolic waste into the parenchyma, triggering microglial activation and white‐matter injury and linking mitochondrial vascular dysfunction to neurodegenerative phenotypes. In fact, recent MRI and CSF biomarker studies indicate that BBB breakdown is detectable early in normal aging and is further accelerated in individuals with MCI, VCI, and early Alzheimer's disease (AD), often preceding overt atrophy or heavy amyloid/tau burden (Qu et al. 2022). Increased BBB permeability allows peripherally derived albumin, fibrinogen, and inflammatory cytokines such as IL‐6 and IL‐8 to enter the brain parenchyma, activating microglia and astrocytes and propagating a neuroinflammatory milieu that damages synapses and oligodendrocytes (Chen et al. 2024). In white‐matter regions, where perfusion is already low and vessel density sparse, this combination of hypoperfusion and barrier leak is thought to be a key driver of small‐vessel disease and VaD (Inoue et al. 2023). Preclinical models in which mitochondrial catalase is overexpressed or mitochondrial‐targeted antioxidants (such as SS‐31 or MitoQ) are administered show improved BBB integrity, reduced neuroinflammation, and better cognitive outcomes, with mitochondria clearly upstream of BBB failure (Tarantini et al. 2018). Recent work provides compelling causal evidence that endothelial mitochondrial metabolism is a primary determinant of BBB stability in aging. Zhan et al. (2023) demonstrated that age‐associated loss of endothelial connexin‐43 reduces intracellular NAD^+^ levels and impairs SIRT3‐dependent mitochondrial function, leading directly to BBB leakage in naturally aged mice. Restoring endothelial NAD^+^ availability, via NMN supplementation or PARP1 inhibition (Tarantini, Yabluchanskiy, et al. 2019), rescued SIRT3 activity, normalized mitochondrial homeostasis, and markedly tightened the BBB, reducing vascular leak and neuroinflammatory signaling (Zhan et al. 2023). These findings position endothelial NAD^+^/SIRT3‐driven mitochondrial integrity not as a downstream consequence of vascular aging, but as an upstream causal node whose preservation can prevent or reverse age‐related BBB failure (Wang et al. 2022). Again, mitochondrial–endothelial dysfunction provides a unifying mechanism. mtROS and peroxynitrite (ONOO^−^) can phosphorylate tight‐junction proteins and cytoskeletal elements, destabilizing junctional complexes and increasing paracellular leak. Senescent ECs accumulate in aged brain microvessels; they exhibit a flattened morphology, altered cytoskeleton, and a pronounced senescence‐associated secretory phenotype (SASP) rich in IL‐1β, IL‐6, TNF‐α, and matrix metalloproteinases that degrade the basement membrane and further compromise BBB integrity (Faakye et al. 2024; Ungvari, Menyhart, et al. 2025). Single‐cell RNA sequencing of aged mouse brain endothelial cells revealed distinct senescent EC subpopulations characterized by mitochondrial dysfunction, elevated ROS, SASP gene expression, and impaired junctional integrity (Kiss et al. 2020). More recently, it was shown that mitochondrial DNA leakage into the cytosol activates the cGAS–STING pathway in vascular aging, leading to NF‐κB–driven inflammatory signaling that further compromises endothelial barrier function (Kim, Kim, and Chung 2023). Genetic clearance of p16Ink4a‐positive senescent cells in mice mitigates age‐related vascular rarefaction and delays organ dysfunction in multiple tissues, including brain and retina (Baker et al. 2011; Lee et al. 2021; Yabluchanskiy et al. 2020), underscoring the pathogenic contribution of senescent ECs and mural cells to NVU failure. Together, these data argue that BBB breakdown in aging is not a passive consequence of “wear and tear,” but an active, mitochondria‐driven process embedded in broader inflammatory and senescence networks (Ungvari, Nyul‐Toth, et al. 2025).

## 4 Time‐Restricted Feeding as a Vascular Rejuvenation Strategy

**FIGURE 2:**
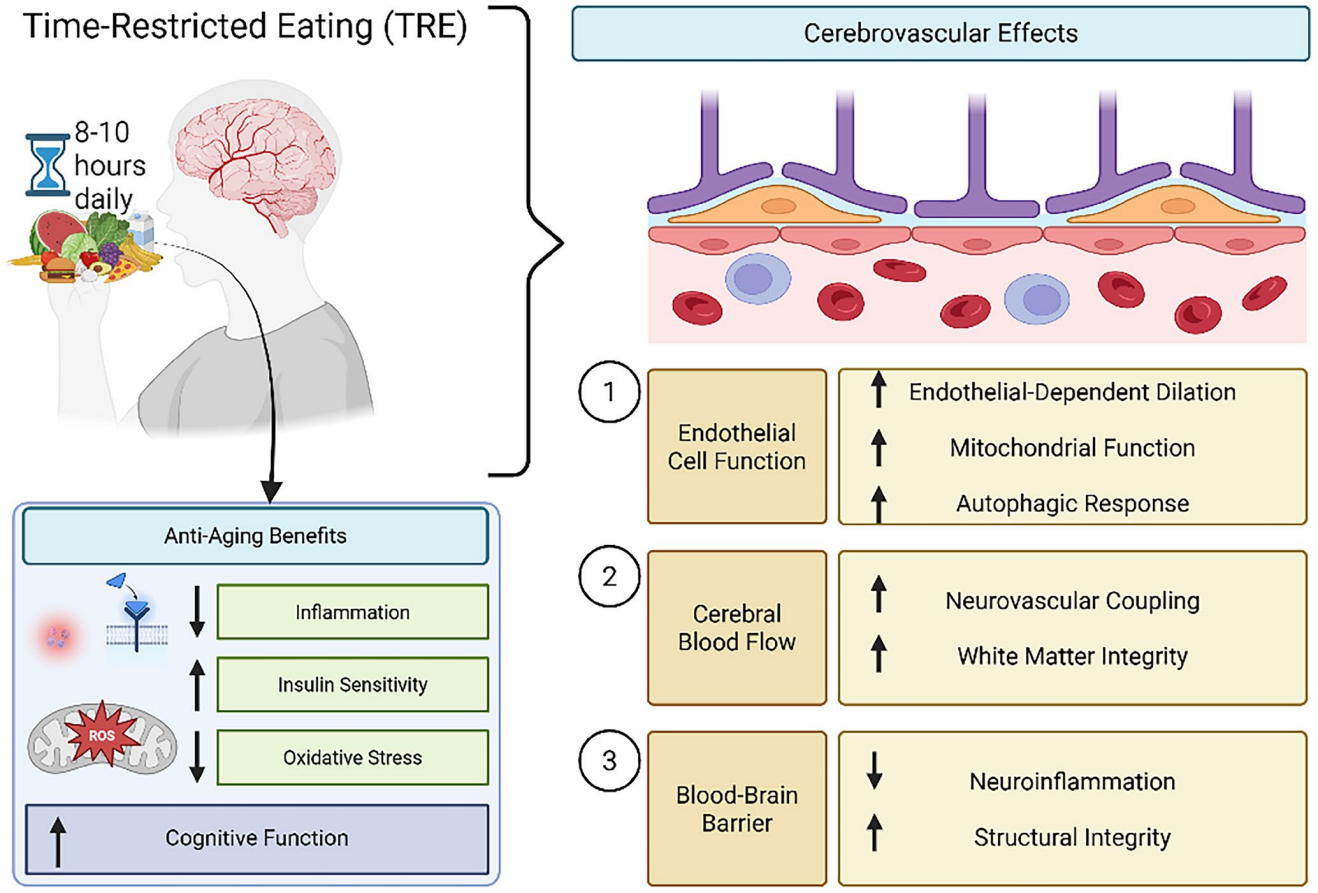
TRF/TRE promotes cerebrovascular resilience and cognitive health through mitochondrial, metabolic, and endothelial mechanisms. Schematic overview of how TRF/TRE, implemented as an 8–10 h daily feeding window, exerts systemic and brain‐specific antiaging effects. TRF/TRE reduces inflammation and oxidative stress, improves insulin sensitivity, and supports cognitive function. At the cerebrovascular level, TRF/TRE enhances EC function by increasing endothelial‐dependent dilation, mitochondrial health, and autophagic activity (1); improves CBF and NVC, contributing to white matter integrity (2); and preserves BBB integrity by reducing neuroinflammation and maintaining structural tight junctions (3). Together, these effects support cerebrovascular function and may protect against age‐related cognitive decline.

TRF/TRE is a form of intermittent fasting (IF) that involves limiting food intake to a specific window of time each day, generally 8–10 h. IF does not involve limiting the number of calories that can be consumed in a day but instead focuses on the amount of time during the feeding window. There are many types of IF, including the 5:2 diet, alternate day fasting, and TRF/TRE. The 5:2 diet includes fasting for 2 days weekly, and AL feeding/eating for the remainder of the week. TRF/TRE allows AL feeding/eating for 6–10 h daily and fasting for the remainder of the day (Di Francesco et al. 2018). TRF/TRE encourages that all calories be consumed within a certain time frame. This diet avoids the potential for malnourishment, frailty, and weakened immune system, while increasing the potential adherence to the dietary regimen as compared to calorie restriction (CR). TRF/TRE may be useful in developing a more approachable intervention for VaD with similar benefits as CR (Figure 2). In a 4‐week study, 90% of participants followed the TRE regimen which included 16 h of fasting daily (Lee et al. 2020). Most participants in this study did not experience uncomfortable hunger, problems with overeating, or decreased energy levels (Lee et al. 2020). Fasting studies have resulted in increased knowledge surrounding the benefits and potential mechanisms of dietary interventions in the context of health and disease. For instance, TRF/TRE has been shown to reduce inflammatory markers, glucose, and insulin (Patterson et al. 2015; Song and Kim 2023). Fasting employs various processes that are known to benefit metabolic functioning. For example, increased antioxidant defenses and regulation of autophagy (de Cabo and Mattson 2019). Animal models that underwent fasting had improved balance and coordination (de Cabo and Mattson 2019). In cardiac tissue, fasting lessens ROS production and improves mitochondrial respiration and mitophagy (Ozcan et al. 2024). Fasting is also a protective factor against atherosclerosis (Ozcan et al. 2024), and improves endothelial‐dependent dilation and angiogenesis (Ozcan et al. 2024). Moreover, it is known that fasting interventions allow for the maintenance of cognition, exhibited by spatial learning and working memory, during aging (de Cabo and Mattson 2019; Mattson et al. 2018).

### 4.1 Nutrient Sensing as a Mechanisms of Time‐Restricted Feeding

**FIGURE 3:**
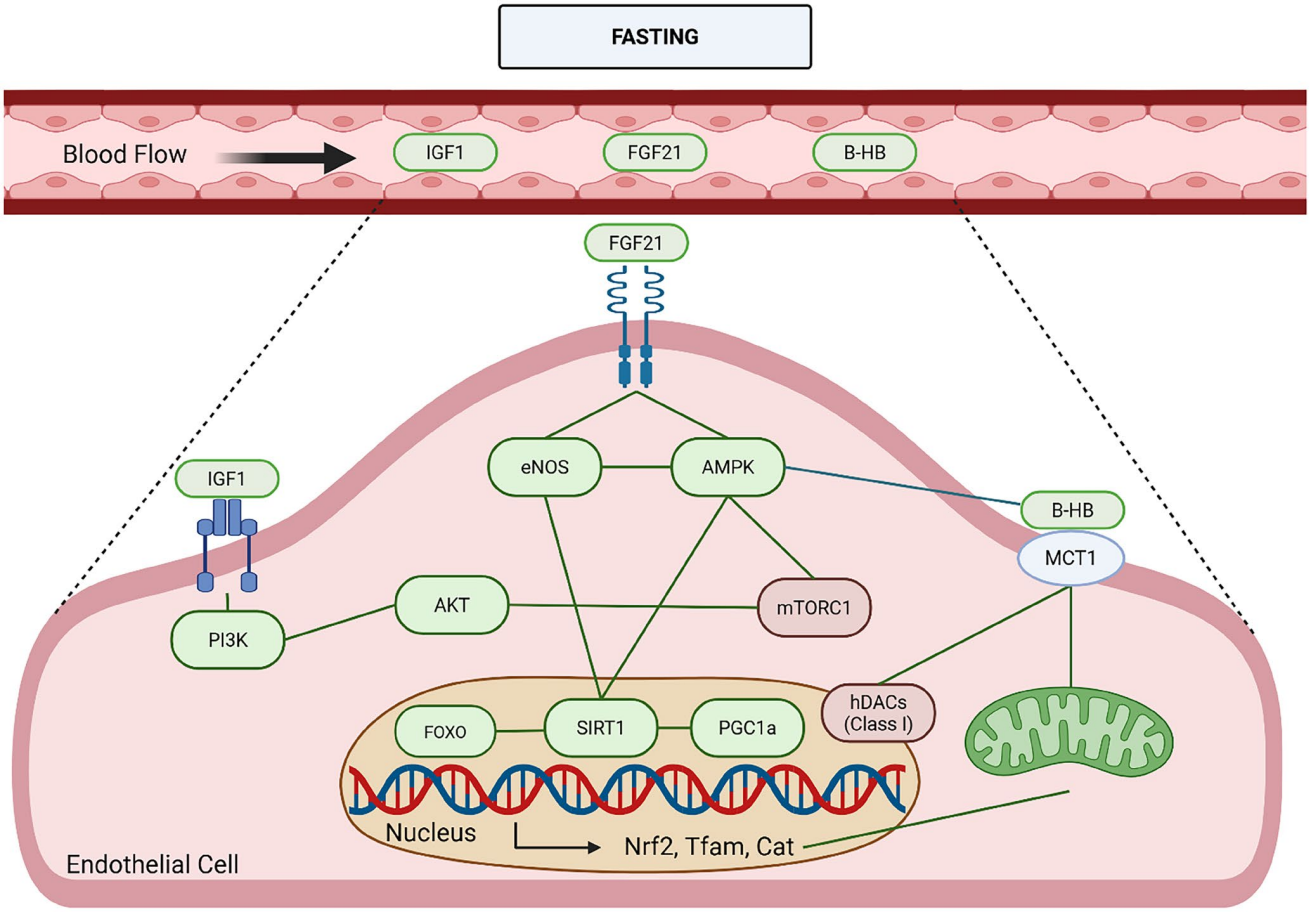
Nutrient‐sensing pathways engaged by fasting and time‐restricted feeding (TRF/TRE) in endothelial cells. Fasting and TRF/TRE induce coordinated endocrine and metabolic adaptations that modulate nutrient‐sensing pathways relevant to cerebrovascular and endothelial health. Systemically, fasting is associated with reduced IGF‐1 signaling, alongside increased circulating fibroblast growth factor 21 (FGF21) and β‐hydroxybutyrate (β‐HB). At the endothelial level, FGF21 and β‐HB signaling engage AMPK, SIRT1, and eNOS, while β‐HB uptake via MCT1 supports mitochondrial metabolism and redox balance. Activation of AMPK and SIRT1 promotes downstream transcriptional programs involving FOXO and PGC‐1α, enhancing antioxidant defenses and mitochondrial biogenesis (e.g., Nrf2, Tfam, and catalase). Concurrently, fasting‐associated attenuation of mTORC1 signaling is linked to reduced pro‐inflammatory and senescence‐associated pathways. Collectively, these pathways are thought to contribute to improved vascular homeostasis and resilience during fasting‐based dietary interventions.

TRF/TRE induces prolonged fasting intervals that activate evolutionarily conserved nutrient‐sensing pathways, including AMPK, SIRT1, and the suppression of mTORC1 (Chaix et al. 2019; Zhang et al. 2024). Activation of AMPK enhances endothelial NO bioavailability (Chen et al. 2009), stimulates mitochondrial biogenesis, promotes autophagy and mitophagy (Rodriguez et al. 2021), and reduces oxidative stress (Jansen et al. 2021). In parallel, the rise in NAD^+^ levels during fasting stimulates SIRT1, which deacetylates eNOS to increase NO production, suppresses inflammatory signaling (Canto et al. 2009; Hoong and Chua 2021), and supports mitochondrial biogenesis through its deacetylation of peroxisome proliferator‐activated receptor γ coactivator‐1α (PGC‐1α) (Wu et al. 2024). The reduction in mTORC1 activity reinforces these processes by alleviating the inhibitory effects of nutrient excess on autophagy and mitochondrial turnover, allowing endothelial cells to efficiently remove dysfunctional mitochondria and maintain metabolic flexibility. Together, these pathways reverse critical aspects of mitochondrial dysfunction and endothelial senescence that characterize vascular aging. Nutrient‐sensing pathways can regulate energy homeostasis, and are very important in aging processes through genes that impact lifespan regulation (Davinelli et al. 2012). There are many relevant pathways, including mammalian target of rapamycin (mTOR), AMP‐activated kinase (AMPK), and sirtuins (Davinelli et al. 2012). mTOR is a serine/threonine protein kinase which has been extensively studied in the context of lifespan regulation, and plays a major role in glycolysis, autophagy, cell growth, and regulation of SASP (Dai et al. 2023; Tomtheelnganbee et al. 2022). More specifically, it has been shown to regulate longevity in *S. cerevisiae* , *C. elegans* , *D. melanogaster* , and *Mus musculus* (Papadopoli et al. 2019; Sharma et al. 2019). It regulates oxygen availability, insulin‐like growth factor 1 (IGF‐1), and glucose (Wu et al. 2022). In addition, mTOR inhibition decreases endothelial senescence in aging, regulates eNOS expression, increases arterial stiffness in aging, and improves endothelial‐dependent dilation in aging (Islam et al. 2024; Wang et al. 2022; Yang, Hou, et al. 2023). It also regulates mitophagy, mitochondrial biogenesis and mitochondrial dynamics (Papadopoli et al. 2019). Fasting can lead to inhibition of mTORC1, which may be responsible for some of the noted benefits (Martens and Seals 2016; Zoncu et al. 2011). AMPK is important for energy metabolism, energy balance, and plays a role in longevity (Salminen and Kaarniranta 2012). AMPK senses mitochondrial stress and nutrient levels, thereby indicating cellular energetics (Wu et al. 2022). It can be activated by increased concentrations of AMP/ADP, as well as specific hormones along with shear stress and exercise (Donato et al. 2015; Salminen and Kaarniranta 2012). In ECs, AMPK is important for angiogenesis and is related to eNOS activation (Donato et al. 2015; Li et al. 2016; Martens and Seals 2016). In aging, there is a reduction in the activation of AMPK. In fact, activation of AMPK using aminoimidazole carboxamide ribonucleotide (AICAR) led to improvement of age‐related EDD dysfunction and oxidative stress (Lesniewski et al. 2012). AMPK has been shown to be activated during TRF/TRE and result in improved health in multiple organisms (Wu et al. 2022). During TRF/TRE, increased AMPK phosphorylation promotes the induction of autophagy (Baherniya et al. 2018) and helps regulate mTORC1 activity (Baherniya et al. 2018). Fasting‐induced AMPK activation can also enhance mitochondrial biogenesis and mitophagy, contributing to improved cellular function (Rodriguez et al. 2021; Sharma et al. 2019). Sirtuins (SIRT) are a family of nicotinamide adenine dinucleotide (NAD+)‐dependent protein deacetylases and ADP‐ribosyltransferases, with 7 different forms in mammals (Martens and Seals 2016). SIRT1, which impacts the growth of blood vessels, can regulate the AMPK pathway and insulin signaling (Izzo et al. 2018). During aging, SIRT1 activity decreases, which can lead to EC dysfunction (Ungvari, Tarantini, Kiss, et al. 2018). SIRT1 deacetylates eNOS in the cytosol to increase NO synthesis (Man et al. 2019; Martens and Seals 2016). SIRT1 also deacetylates FOXOs and alters transcription of NFkB, ultimately leading to a decrease in oxidative stress and may be essential for the observed lifespan extension in organisms on CR (Martens and Seals 2016; Wu et al. 2022; Yang, Velagapudi, et al. 2023). SIRT1 and 3 are known to be activated during fasting, and have been linked to improvements in mitochondrial biogenesis and decreased senescence (Hammer et al. 2021; Madkour et al. 2019; Zhu et al. 2013; Zu et al. 2010). In humans practicing TRE with the eating period during morning hours, SIRT1 was upregulated during this period (Jamshed et al. 2019). Additionally, when CR is employed, SIRT1 increases in rats, human cell culture, and human tissue (Wu et al. 2022). Activation of SIRT1 by nicotinamide mononucleotide (NMN) intervention ameliorates age‐related endothelial dysfunction in mice (de Picciotto et al. 2016). Further, supplementation with NMN during aging promotes the maintenance of NVC responses and working memory (Tarantini, Valcarcel‐Ares, et al. 2019). Each of these mechanisms of nutrient‐sensing pathways can be altered during aging, and they play important roles in the regulation of longevity. Modification of these pathways resulting from pharmacological treatments, supplementation, or dietary restriction may be responsible for some antiaging effects (Figure 3).

### 4.2 Metabolic Switching as a Mechanism of Time‐Restricted Feeding/Eating

During periods of fasting, the body can undergo a metabolic switch that can be characterized as a change in the utilization of different fuel sources. Fasting results in a metabolic switch toward using fatty acids and ketones rather than glucose, particularly in muscle, heart, and brain (Anton et al. 2018; Han et al. 2020). After complete utilization of hepatic glycogen stores during fasting, lipids are converted into free fatty acids (FFAs) and transported through the blood (Anton et al. 2018). FFAs can further be converted into ketone bodies like beta‐hydroxybutyrate (β‐OHB) and acetoacetate (AcAc) through β‐oxidation (Anton et al. 2018). As a product of fatty acid oxidation, AcAc can further be converted to acetone, or to β‐OHB by β‐hydroxybutyrate dehydrogenase (BDH1) (Newman and Verdin 2014). It is known that ketogenic diets, CR, and ketone ester supplementation can increase levels of circulation β‐OHB (Han et al. 2020; Lin et al. 2015). β‐OHB cannot readily cross the BBB; thus, it requires monocarboxylic acid transporters such as MCT1 and MCT2 (Leino et al. 2001; Newman and Verdin 2014). Differentially expressed genes during fasting include solute carrier (SLC) transporters like GLUT1, MCT1, and SNAT3, which are responsible for the transport of glucose, ketone bodies, and glutamine, respectively (Chasseigneaux et al. 2024). Increases in brain β‐OHB have many downstream effects, including increased expression of brain‐derived neurotrophic factor (BDNF) in hippocampus and cortex, which is important for synaptic transmission and plasticity (specifically in long‐term potentiation), with a key role in adult neurogenesis, learning, and memory (Garcia‐Rodriguez and Gimenez‐Cassina 2021; Mattson et al. 2018). In addition, ketone bodies can be utilized for cell signaling, even increasing angiogenesis and cell proliferation in cardiac ECs (Lopaschuk et al. 2022; Weis et al. 2022). β‐OHB can inhibit histone deacetylases (HDACs), which can result in decreased neuroinflammation and increased expression of antioxidant genes Foxo3a and Mt2 (Garcia‐Rodriguez and Gimenez‐Cassina 2021; Han et al. 2020; Lopaschuk et al. 2022). β‐OHB also prevents senescence in ECs and VSMCs by binding to heterogeneous ribonuclear particle A1 (hnRNP A1) to increase Oct4A, reducing IL‐1α (Han et al. 2018; Nasser et al. 2020). The effects of β‐OHB are highly connected to changes in nutrient‐sensing pathways. Ketogenesis is limited by 3‐hydroxy‐3‐methylglutaryl‐CoA synthase 2 (HMGCS2), which is transcriptionally regulated by FOXA2 and the mTORC1 pathway (Newman and Verdin 2014). FOXA2 is strongly affected by changes in insulin signaling, SIRT1, and classes I and II HDACs (Newman and Verdin 2014). mTORC1 also modulates the effect of ketone bodies through the regulation of peroxisome proliferator‐activated receptor alpha (PPARα) and fibroblast growth factor (FGF21) (Newman and Verdin 2014). Therefore, the interaction of metabolic switching and multiple nutrient‐sensing pathways during fasting interventions may be contributing to many downstream benefits including improvements of vascular and mitochondrial function in aging.

### 4.3 Circadian Alignment and Restoration of Vascular Rhythmicity

TRF/TRE also functions as a potent synchronizer of metabolic and vascular rhythms that become progressively misaligned with age. Endothelial cells, mitochondria, and nutrient‐sensing pathways follow intrinsic circadian oscillations that regulate NO production, antioxidant defense, mitochondrial respiration, and vascular tone. With age, these rhythms lose amplitude and precision (Thosar et al. 2018). Further, disruption of circadian alignment in vascular tissue results in attenuated daily variations in NO production and loss of the normal amplified vascular responses in the morning that aid transition from sleep to activity (Krilis et al. 2025). TRF/TRE restores temporal coherence by restricting food intake to metabolically appropriate periods of the day, thereby reinforcing the transcriptional programs that govern mitochondrial function and endothelial health. Human studies using early TRE (calorie consumption in morning hours) demonstrate improvements in insulin sensitivity, blood pressure regulation, oxidative stress profiles, and inflammatory markers, all of which provide a systemic environment conducive to vascular rejuvenation (Krilis et al. 2025; Maury et al. 2010; Petridi et al. 2024).

### 4.4 Metabolic Switching and Ketone‐Mediated Vasoprotection

A defining feature of TRF/TRE is the induction of a metabolic switch from glucose‐dominant metabolism to fatty acid oxidation and ketone body utilization. The ketone body β‐hydroxybutyrate (β‐OHB) is not only a bioenergetic substrate but also a signaling molecule. During metabolic switching, β‐OHB becomes the predominant ketone body produced from hepatic ketogenesis during periods of fasting or low carbohydrate availability, representing approximately 70% of circulating ketone bodies (Achanta and Rae 2017). Within mitochondria, β‐OHB undergoes oxidation to generate acetyl‐CoA through β‐OHB dehydrogenase (BDH1), entering the tricarboxylic acid cycle to support oxidative phosphorylation (Kadir et al. 2023). This metabolic pathway exhibits a defining characteristic: the direct oxidation of β‐OHB alters the NAD^+^/NADH and ubiquinone/ubiquinol redox couples in ways distinct from glucose oxidation, resulting in a more negative redox potential within the NADPH antioxidant system (Veech et al. 2017). Consequently, mtROS generation is substantially reduced compared to glucose‐dependent metabolism (Holstein et al. 2025). This shift toward lower ROS production occurs through multiple mechanisms, including enhanced electron transport chain efficiency, reduced Complex I‐dependent reverse electron transport, and improved mitochondrial coupling efficiency (Gomora‐Garcia et al. 2023). The enhanced mitochondrial membrane potential and reduced proton leak achieved through β‐OHB oxidation strengthen the energetic foundation upon which endothelial cells maintain their stringent ionic gradients and synthesize the ATP required for continuous tight junction protein maintenance and vascular tone regulation. The consequence is widespread upregulation of the endogenous antioxidant capacity that endothelial cells require to preserve NO bioavailability and prevent the oxidative scavenging of NO (Kolb et al. 2021). Additionally, β‐OHB‐mediated HDAC inhibition activates sirtuins, particularly SIRT1 and SIRT2, which themselves function as NAD^+^‐dependent protein deacetylases that regulate mitochondrial biogenesis, mitochondrial quality control through mitophagy, and autophagy (Gomora‐Garcia et al. 2023). β‐OHB enhances histone 3 lysine 9 hydroxybutyrylation at the tight‐junction protein 1 (TJP1) gene promoter, driving transcriptional upregulation of this essential tight junction component (Li, Liu, et al. 2024). In the context of cardiac repair, β‐OHB specifically targets macrophages to stimulate vascular endothelial growth factor (VEGF) production in periinfarction regions through a mechanism involving enhanced histone acetylation of genes controlling VEGF transcription (Wang, Xu, et al. 2025). At the cellular level, β‐OHB attenuates endothelial cell senescence through multiple coordinated mechanisms. Senescent endothelial cells display accumulated p16, p21, and p53 expression, reduced SIRT1 levels, and impaired endothelium‐dependent nitric oxide production (Viswambharan et al. 2007). Treatment with β‐OHB reverses these hallmarks by activating SIRT1‐dependent deacetylation of eNOS, restoring NO production capacity (Gomora‐Garcia et al. 2023). Furthermore, β‐OHB suppresses the senescence‐associated secretory phenotype (SASP) by reducing the release of pro‐inflammatory cytokines and chemokines that would otherwise activate surrounding cells and promote chronic inflammation (Jin et al. 2023). This anti‐senescence action extends to smooth muscle cells within the medial layer of blood vessels; β‐OHB prevents the phenotypic switch from contractile to synthetic smooth muscle cells that typically accompanies vascular aging and atherosclerosis (Ji et al. 2022).

### 4.5 TRF/TRE and Improvement of Vascular, Neurovascular, and BBB Function

The molecular actions of TRF/TRE translate into robust improvements in vascular physiology across multiple domains. TRF/TRE restores endothelial‐dependent dilation by enhancing NO signaling and reducing ROS, while concurrently reducing arterial stiffness and improving hemodynamic regulation. In a rat model of high‐fat diet‐induced obesity, 6 weeks of TRF significantly improved endothelium‐dependent relaxation to acetylcholine while reducing endothelium‐dependent contraction, improvements that were associated with increased aortic eNOS and Akt protein expressions (Azemi et al. 2022). These findings demonstrate that TRF/TRE restores the fundamental vasodilatory capacity of ECs through enhanced NO pathway signaling. Recent research has established that NO plays an essential role in NVC in humans, with NO synthase blockade reducing NVC responses by approximately 30% (Rocha et al. 2022). Six months of TRF in aged mice markedly improved endothelial‐dependent dilation of aortic tissue while simultaneously increasing oxygen consumption and reducing ROS production in aortic tissue (Milan et al. 2024). Six weeks of 10‐h eating windows with 14‐h fasting periods significantly reduced pulse wave velocity (PWV; a marker of arterial stiffness by 0.29 m/s), decreased augmentation index (reduction of 4.03 mmHg), and improved central systolic pressure (reduction of 2.43 mmHg) (Alinezhad‐Namaghi et al. 2023). These improvements in central hemodynamic parameters indicate that TRF/TRE ameliorates arterial stiffening that characterizes aging and metabolic disease. In the brain, TRF/TRE improves NVC, augments CBF, and preserves microvascular density. A pilot study in older adults demonstrated that 6 months of 10:14 intermittent fasting (10‐h eating window, 14‐h fasting) was associated with significant increases in NVC responses in the dorsolateral prefrontal cortex, the region relevant to cognitive stimulation (da Langley et al. 2024). TRF/TRE was associated with increased levels of tetrahydrofolate, L‐arginine, and nicotinamide, metabolites involved in vascular health, as well as an increase in several metabolites directly implicated in antioxidant, anti‐inflammatory, and anti‐senescence pathways in the vasculature (da Langley et al. 2024).

## 5 Translational and Clinical Implications of TRF/TRE in Cognitive Aging

### 5.1 Feasibility, Safety, and Metabolic Benefits in Humans

Human studies indicate that TRE is practical, well‐tolerated, and safe across diverse populations, including older adults with cardiometabolic disease (Balasubramanian et al. 2020; James et al. 2024; Lee et al. 2020). Participants readily adopt consistent feeding windows and often maintain high adherence over extended periods. TRF/TRE improves longevity and autophagy‐related gene expression, glucose regulation, insulin resistance, blood pressure, lipid handling, circadian alignment, and systemic inflammation, even without weight loss (Chang et al. 2024; Jamshed et al. 2019; Manoogian et al. 2022; Moro et al. 2021; Steger et al. 2023; Sutton et al. 2018; Xie et al. 2022). These improvements directly influence cerebrovascular risk by reducing endothelial oxidative stress, limiting metabolic injury to the vasculature, and improving systemic hemodynamic stability.

### 5.2 Implications for Cerebrovascular Aging and Cognitive Decline

To date, no large, prospective, randomized controlled trial has assessed whether TRF/TRE delays cognitive decline or reduces the incidence of mild cognitive impairment (MCI) or dementia in cognitively normal older adults. Existing human TRF/TRE studies have focused on metabolic outcomes (weight loss, glucose control, lipid profiles, and blood pressure) rather than cognitive or cerebrovascular function (Cena and Calder 2020). It is plausible that given the above illustrated mechanisms, TRF/TRE holds strong implications for cerebrovascular aging and cognitive improvement. By rejuvenating endothelial mitochondria and restoring NO signaling, TRF/TRE reduces pulsatile stress on the cerebral microcirculation and enhances the ability of vessels to match blood flow to neural activity. Improvements in mitochondrial quality control and suppression of inflammatory signaling preserve microvascular structure, maintain NVC, and support cerebral perfusion. TRF/TRE also strengthens BBB integrity, decreasing endothelial permeability and limiting the entry of circulating inflammatory and neurotoxic factors into the brain. Recent human studies support the translational potential of these mechanisms. A 6‐month TRE intervention (10‐h eating window) in community‐dwelling older adults (aged 55–80 years) demonstrated improvements in peripheral endothelial function and is currently being evaluated for its effects on NVC responses and cognitive performance (Pinaffi‐Langley et al. 2024). Furthermore, a 4‐month TRE regimen in AD patients ameliorated cognitive impairments through mechanisms involving gut microbiota modulation and enhanced propionic acid production, which subsequently improved neurovascular and neuroinflammatory parameters (Zhao et al. 2025). In metabolic syndrome populations, 12 weeks of TRE (10‐h eating window) resulted in significant reductions in systolic blood pressure (4.8 mmHg), improved glycemic control, and decreased inflammatory markers, all of which are established risk factors for VCI (Swiatkiewicz et al. 2024). These human data complement preclinical findings showing that TRF/TRE enhances CBF regulation, strengthens BBB integrity, and improves cognitive function through endothelial‐dependent mechanisms. The convergent actions from both mechanistic and clinical studies point toward TRF/TRE as a promising, cost‐effective, and feasible strategy for delaying VCI, cerebral small vessel disease, and mixed dementia pathologies.

### 5.3 Considerations for Clinical Implementation Across the Aging Population

Translating TRF/TRE into widespread clinical practice, particularly within the diverse aging population, requires careful and strategic optimization that moves beyond a one‐size‐fits‐all approach. While preclinical evidence strongly supports the mechanisms by which TRF/TRE can benefit cardiovascular and brain health, ongoing research is crucial to establish efficacy, safety, and optimal protocols in humans (Kim et al. 2022). Evidence to date suggests that earlier feeding windows (eTRF) may be more effective in reinforcing endogenous circadian and metabolic rhythms than late‐day schedules, aligning food intake with the body's natural metabolic peaks and troughs (Dibner and Schibler 2015; Panda 2016). This chronobiological alignment is particularly important in older adults, who often experience dampened circadian rhythms that contribute to age‐related cardio‐metabolic and neurological dysfunction (Thosar et al. 2018). However, the efficacy and safety of TRF/TRE are not universal across all individuals and necessitate careful individualized adaptation. Factors such as sex differences, baseline metabolic state, and existing comorbidity profiles significantly influence how an individual responds to TRF/TRE (Manoogian et al. 2022). For instance, preclinical studies highlight sex‐specific metabolic responses to fasting protocols. This underscores the importance of investigating nutritional interventions in both males and females (Bradshaw et al. 2024; Fishbein et al. 2021; Munoz et al. 2020). Older adults often present with multiple comorbidities, conditions like sarcopenia, may require careful protein intake optimization alongside TRF/TRE to prevent muscle loss, emphasizing the need for protein‐rich meals during the eating window and potentially adapted fasting durations (Kim, Jung, and Williams 2023). Combining TRF/TRE with other interventions that target complementary mechanisms may potentiate its benefits and broaden its applicability, addressing the multifactorial nature of aging. For example, synergistic effects are anticipated when TRF/TRE is combined with aerobic exercise, as both interventions independently activate AMPK and SIRT1 pathways, promote mitochondrial biogenesis, and enhance endothelial function (Donato et al. 2018), leading to improved cardiovascular health (Clayton et al. 2022). Similarly, the use of NAD^+^ precursors (such as NR or NMN) could complement TRF/TRE by directly replenishing NAD^+^ levels critical for sirtuin activation and mitochondrial function, potentially amplifying the cellular rejuvenation effects (Bita et al. 2023; Diaz‐Ruiz et al. 2018). Senolytic therapies, aimed at selectively clearing senescent cells, represent another promising avenue for combination, as cellular senescence is a hallmark of aging implicated in vascular dysfunction (Le 2023; Suda et al. 2024).

### 5.4 Outlook for TRF/TRE as a Geroscience‐Based Cerebrovascular Intervention

To fully establish TRF/TRE's therapeutic potential in an aging population, future clinical trials must incorporate comprehensive phenotyping. This should include advanced vascular imaging (e.g., flow‐mediated dilation, pulse wave velocity to assess arterial stiffness) to directly measure improvements in endothelial function and vascular health (Clayton et al. 2022; Li et al. 2023). Assessment of endocrine markers (e.g., insulin sensitivity, adipokines, and sex hormones) and mitochondrial function assays (e.g., mitochondrial respiration, biogenesis markers in accessible tissues) will provide critical mechanistic insights into the systemic and cellular responses to TRF/TRE (Smith et al. 2018; Veluthakal et al. 2024). Crucially, cognitive assessment using sensitive and validated batteries will be essential to demonstrate direct neuroprotective effects and translate metabolic benefits into meaningful cognitive outcomes. This multi‐modal assessment will enable better stratification of responders, optimize personalized TRF/TRE protocols, and integrate TRF/TRE into broader lifestyle medicine strategies for healthy aging (Clemente‐Suarez et al. 2023; Vodovotz et al. 2020). Although more longitudinal research is needed, TRF/TRE stands out as an accessible, non‐pharmacological, and mechanistically grounded strategy capable of addressing the proximal causes of vascular and cognitive aging. By restoring mitochondrial integrity, improving endothelial function, and reinforcing the stability of the NVU, TRF/TRE may represent a pivotal tool for preserving cerebrovascular health and cognitive performance across the lifespan. Its capacity to reactivate intrinsic cellular resilience pathways positions TRF/TRE as a promising candidate for integration into preventive neurology and healthy aging frameworks worldwide.

## 6 Conclusions

Vascular aging is not a peripheral consequence of growing old but a central, organizing pathology that links systemic metabolic decline to the failure of cerebral homeostasis. At the core of this process lies a progressive collapse of mitochondrial function within endothelial cells, which erodes redox balance, disrupts nitric oxide signaling, drives cellular senescence, and compromises the structural and functional integrity of the neurovascular unit. Because the brain depends on exquisitely regulated perfusion and barrier function to sustain cognition, even subtle decrements in endothelial mitochondrial health propagate through the microvascular network, producing neurovascular uncoupling, BBB breakdown, and vulnerability to both Alzheimer's disease and vascular dementia. Mounting evidence places this mitochondria–endothelium axis at the epicenter of vascular cognitive impairment, positioning mitochondrial dysfunction not as an accompanying feature but as a causal determinant of cerebrovascular aging. TRF/TRE offers a compelling strategy to counter these age‐related changes by restoring mitochondrial efficiency, reducing oxidative stress, re‐engaging mitophagy and biogenesis. By realigning nutrient intake with intrinsic circadian rhythms and inducing regular cycles of metabolic switching, TRF/TRE activates a set of nutrient‐sensing pathways (AMPK and SIRT1), and the suppression of mTORC1 that restore mitochondrial quality control, enhance oxidative phosphorylation, promote mitophagy, and reduce endothelial inflammation and senescence. These effects collectively strengthen vascular tone, improve NVC, reinforce BBB integrity, and preserve cerebral perfusion. Importantly, TRF/TRE achieves this without requiring caloric restriction, underscoring its ability to reactivate ancient cellular resilience programs rather than impose metabolic deprivation. Across animal models, TRF/TRE consistently rejuvenates endothelial and cerebrovascular function; early human studies suggest that these mechanisms may translate to older adults. As the burden of dementia continues to rise worldwide, strategies capable of protecting the aging cerebral microvasculature are urgently needed. TRF/TRE stands out as a feasible, low‐cost, mechanistically grounded intervention that directly targets the mitochondrial defects driving vascular and cognitive decline. Future work integrating advanced imaging, endothelial‐specific biomarkers, and longitudinal cognitive outcomes will be critical to establish TRF/TRE's efficacy and to identify the individuals most likely to benefit. Nevertheless, the convergence of geroscience, vascular biology, and nutritional physiology now supports a compelling model: by restoring mitochondrial integrity within the endothelium, TRF/TRE has the potential to preserve cerebrovascular health, delay cognitive aging, and extend the healthy lifespan.

## Author Contributions

All the authors of this manuscript have contributed significantly to the article.

## Funding

This work was supported by the American Heart Association (CDA941290, 25IPA1456700, 24TPA1299954, 25POST1377493, and 24DIVSUP1280258) and National Institute on Aging (K01AG073614, K01AG073613, R03AG070479, and R21AG080775).

## Conflicts of Interest

The authors declare no conflicts of interest.

## Data Availability

Data sharing not applicable to this article as no datasets were generated or analysed during the current study.
